# Barriers to Cervical Cancer Screening and the Cervical Cancer Care Continuum in Rural Guatemala: A Mixed-Method Analysis

**DOI:** 10.1200/JGO.17.00228

**Published:** 2018-06-15

**Authors:** Kirsten Austad, Anita Chary, Sandy Mux Xocop, Sarah Messmer, Nora King, Lauren Carlson, Peter Rohloff

**Affiliations:** **Kirsten Austad**, **Anita Chary**, **Sandy Mux Xocop**, **Sarah Messmer**, **Nora King**, and **Peter Rohloff**, Wuqu’ Kawoq, Maya Health Alliance, Santiago Sacatepéquez, Guatemala; **Kirsten Austad**, Brigham and Women’s Hospital, Boston, MA; and **Lauren Carlson**, Mayo Medical School, Minnesota Campus, Rochester, MN.

## Abstract

**Purpose:**

Cervical cancer is an important cause of mortality in low- and middle-income countries. Although screening technologies continue to improve, systems of care remain fragmented. It is important to better understand factors that affect use of screening services and loss to follow-up along the care continuum.

**Methods:**

We conducted a mixed-methods study of a cytology-based screening program in rural Guatemala. A retrospective electronic chart review was performed on data from all patients from 2013 to 2014. We analyzed progression through care and calculated loss–to–follow-up rates. We also analyzed the prior experiences of patients with cervical cancer screening on the basis of self-reported historical data available in the chart review. Structured interviews with a subset of individuals to explore social supports and barriers to screening and engagement in care were conducted at the time of screening.

**Results:**

The analysis included 515 women (median age, 36 years). Cytologic screening showed concern for neoplastic changes in 0.83%; half resulted in biopsy-proven cervical intraepithelial neoplasia. An additional 9.9% showed severe inflammation. The rate of loss to follow–up was 11.3%. All losses to follow-up occurred for severe inflammation, not for cervical intraepithelial neoplasia. Historical data showed that 73% of the cohort had previously been screened and had high levels of loss to follow-up (57.4%). Qualitative interviews revealed factors that promoted loss to follow-up; these included cost, lack of social supports, transportation, distrust in public facilities, long turn-around times, and failure to return test results or offer follow-up treatments.

**Conclusions:**

Taken together, these quantitative and qualitative results highlight the need for cervical cancer screening programs in Guatemala to improve uptake of screening services by eligible women and to improve follow-up after a first abnormal screen.

## INTRODUCTION

Worldwide, cervical cancer affects primarily poor populations, and more than 85% of cervical cancer deaths occur in low- and middle-income countries (LMICs).^1,2^ In Guatemala, the most populous country in Central America, 13% to 33% of women are infected with human papilloma virus (HPV),^3-5^ and cervical cancer is the most common cancer in women younger than age 50 years.^6,7^

In high-income countries, screening programs that use cervical cytology (Papanicolaou) have reduced the incidence of cervical cancer. However, cytology-based programs have been less effective in LMICs.^8-10^ This is due to the resource-intensive demands of screening and the inefficiencies along the continuum of care that cause loss to follow-up (LTFU).^9-11^

The global oncology community has worked to improve cervical cancer detection through new screening technologies, including visual inspection with acetic acid (VIA) and HPV-based molecular detection.^12,13^ In 2013, WHO recommended that HPV-based screening become a first-line approach, and many LMICs are transitioning toward HPV-based techniques.^14^ Guatemala incorporated HPV testing into national guidelines in 2015.^15^

However, compared with the interest in screening technologies, there has been less effort to understand the cervical cancer continuum of care and the barriers to screening and follow-up.^16-20^ This is a gap in knowledge, because multiple factors along the continuum of care affect the effectiveness of screening programs, regardless of the underlying technology.^21^

Knowledge of the cervical cancer care continuum in Guatemala, as in many LMICs, is partial, because care is provided by a diverse array of community-based and referral institutions; there is no centralized record keeping and little coordination. Here, we present clinical data from one community-based cervical cancer screening program in rural Guatemala with an electronic medical record (EMR) system, which permitted tracking of outcomes. We complement this analysis with qualitative interviews from a subset of patients to explore barriers and facilitators to screening and follow-up care. The overall goal of this analysis is to respond to recent calls for system-based analyses of women’s cancers in LMICs^22^ by providing a preliminary analysis of the cervical cancer care continuum.

## METHODS

### Institutional and Program Description

The study was conducted at Maya Health Alliance (MHA), a primary care organization that serves indigenous Maya communities in Guatemala. MHA expanded cervical cancer screening services in 2013 to 14 villages in the central Guatemalan provinces of Chimaltenango and Suchitepéquez. Nurses provide mobile door-to-door screening services at no cost. Women age 21 years and older are offered cytology-based screening and receive individualized counseling and a standardized clinical interview.

**Fig A1:**
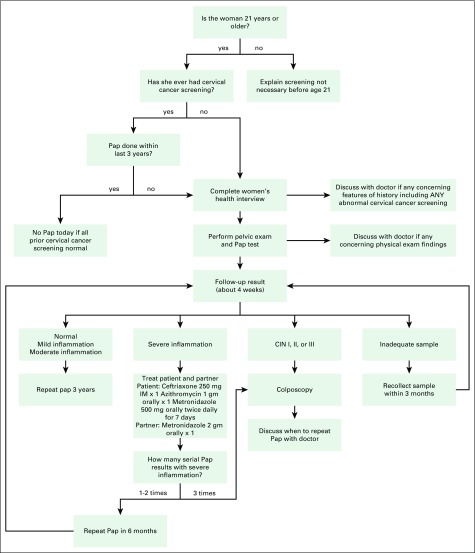
Management algorithm for cervical cytology results. The image depicts the clinical workflow used by the partnering institution in Guatemala (Maya Health Alliance) to manage cervical cytology results of women without a history of abnormal cervical cancer screening. This algorithm allows field nurses to determine the appropriate treatment plan and screening intervals for most clinical cases. For patients with a history of cervical intraepithelial neoplasia (CIN), cervical procedures, or other high-risk features, a doctor formulates an individualized treatment plan. Partner treatment indicates differences from patient treatment (ie, partners treated with same dose of ceftriaxone and azithromycin, only dose of metronidazole changes).

Cytology specimens are analyzed by the reference laboratory at the Instituto de Cancerología (INCAN, Institute for Cancer). As in many LMICs, this laboratory does not follow standardized international reporting guidelines.^23^ Consequently, MHA staff use clinical algorithms that are based on guidelines from the American Society for Colposcopy and Cervical Pathology for management (Appendix Fig A1).^24^ INCAN reports often comment on the degree of inflammation, and severe inflammation is of particular concern, because it may indicate HPV infection and mask early neoplastic changes.^25,26^ Individuals with severe inflammation are treated empirically for sexually transmitted infections (STIs) and undergo repeat cytology testing (Appendix Fig A1).

Women who require colposcopy for possible neoplastic cells, or who have severe inflammation that persists after STI treatment, are referred to INCAN, which provides publicly subsidized care to poor patients. Throughout the referral, an MHA care navigator interacts with INCAN staff, which permits tracking of progress through treatment or LTFU.^27^ All clinical data are recorded in an EMR (OpenMRS, http://www.openmrs.org).

### Ethics

Study protocols for the EMR review were approved by the institutional review boards of MHA (WK-2015-004) and Partners Healthcare (2015P001346). Study protocols for the qualitative interviews were approved by the Washington University in St. Louis institutional review board (201203043).

### Quantitative Analysis

We conducted a retrospective EMR review of patients who presented for screening from the beginning of the MHA program in February 2013 through December 2014. This review was part of a programmatic review, as part of a quality improvement initiative and planning for scale-up. All women age 16 to 75 years with an intake form in the EMR were included. From this form, clinical data were abstracted by three study authors (S.M., L.C., and K.A.); data included demographics, prior cervical cancer screening (self-reported results), family planning use, and obstetric history (full variable list provided as a Data Supplement). In addition, results of cervical cytology and follow-up care performed by MHA were abstracted. The data set was checked for accuracy by comparing abstracted information with that of the EMR (L.C., K.A.). We performed statistical analyses in STATA, version 14 (College Station, TX). Continuous variables were summarized as medians with interquartile ranges, because most were nonparametric, and categoric data were summarized as percentages.

### Qualitative Analysis

As part of an early-stage implementation assessment of the MHA program, women who attended cervical cancer screening clinics in March 2013 were visited at home to solicit a structured interview. Overall, 54 women were home at the time of the visit, and all accepted the interview. One author (A.C.) conducted interviews in the patient’s preferred language (Spanish, Kaqchikel, or K’iche’), with the assistance of an interpreter for indigenous languages. Interviews explored knowledge of cervical cancer, indications for screening, personal motivations, and barriers and facilitators to screening and treatment (interview guide provided in the Data Supplement). Given the sensitive nature of the topics, we documented interviews with note taking.

We extracted interview responses into spreadsheets and checked field notes for accuracy. Two authors (K.A. and A.C.) organized data into dominant themes and chose representative quotes to illuminate selected themes. Synthesized data were reviewed by a third author (P.R.).

## RESULTS

### Characteristics of Patients Who Seek Cervical Cancer Screening

We identified 515 patients who had cervical cancer screening intake forms in the EMR. These included 15 women age 16 to 20 years (n = 15) who were not eligible for screening (Appendix Fig A1) but who are included in the baseline descriptive analysis (Table 1).

**Table 1:**
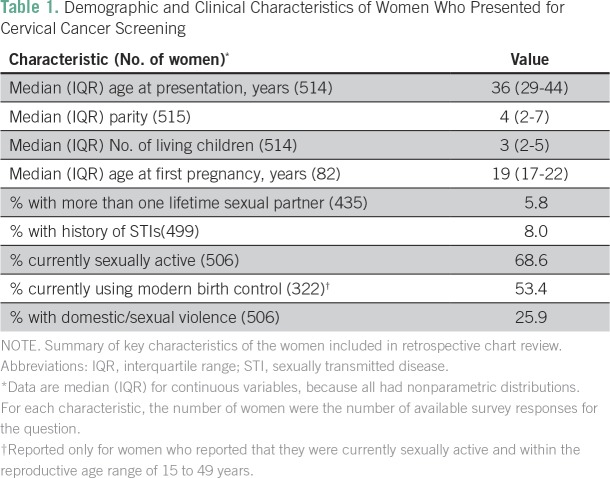
Demographic and Clinical Characteristics of Women Who Presented for Cervical Cancer Screening

Among women who were sexually active and responded to questions about birth control, 53.4% were using a form of modern birth control, including depot medroxyprogesterone acetate (30.4%), surgical sterilization (15.6%), condoms (2.6%), and combined oral contraceptives (2.0%). Only nine women (2.8%) were users of long-acting reversible contraception. In addition, many women reported gynecologic symptoms, including vaginal discharge (31.3%), vaginal itching (19.4%), burning with urination (10.6%), and pain with intercourse (13.4%). Overall, 8.0% reported a previous STI. A significant number (25.9%) reported a history of domestic or sexual violence.

### Results of Screening and Follow-Up Care

Five hundred of the 515 women identified in the EMR were eligible for screening. Of those, 96.6% (n = 483) underwent cervical cytology screening, and 89.0% had normal results (Table 2). Four patients (0.83%) had atypical cells that were concerning for malignancy. All four underwent colposcopy with biopsy: two showed cervicitis (resolved after STI treatment), and two had confirmed cervical intraepithelial neoplasia (CIN) III; thus, the final prevalence of biopsy-proven premalignant lesions was 0.41%. Both individuals were referred to INCAN. One underwent cone excisional biopsy with clear borders, and the other opted for hysterectomy.

**Table 2:**
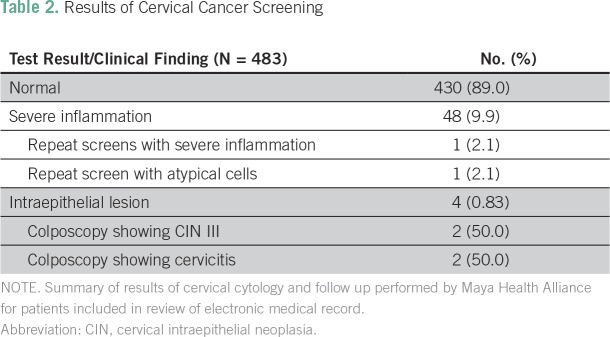
Results of Cervical Cancer Screening

In addition, 9.9% of individuals had severe inflammation without atypia. Per protocol (Appendix Fig A1), they and their partners were treated for STIs. On repeat screening, two women demonstrated persistent abnormalities, one with severe inflammation and the other with atypical cells that were concerning for malignancy. Both underwent colposcopy with biopsy, and no premalignant lesions were found.

### Cervical Cancer Care Cascade and Loss to Follow-Up

We analyzed the cervical cancer care cascade for both (1) self-report of prior cervical cancer screening and treatment (from the medical history section of the EMR form) and (2) documented screening and follow-up care provided by MHA. This allowed us to visualize weak points in care both before and within the MHA system.

#### Recall history of screening and follow-up care.

**Fig 1:**
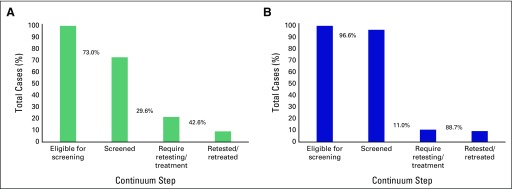
Continuum of care for cervical cancer screening and treatment. The graphics depict the proportion of individual women that successfully reach each of the four steps in the care continuum, beginning with the population eligible for screening in the leftmost column (100% by definition) and ending with the proportion of women with abnormal results who were successfully retested or retreated after an abnormal result. (A) Prior screening activities, which were based on self-report during the initial clinical interview. (B) Results of current screening performed by Maya Health Alliance, as abstracted from the electronic medical record. Percentages between bars represent the proportion of women who advanced to each step in care relative to the prior step.

According to self-reported data (Table 3; Fig 1A), only 73% of individuals, all of whom were eligible for screening on the basis of their age at time of the encounter, reported screening before MHA care. Among the 29.6% (108 of 365 women) who reported an abnormal screen, only 42.6% reported follow-up testing or treatment (46 of 108 women). This resulted in an effective LTFU of 57.4% for retesting or treatment of abnormal test results.

**Table 3:**
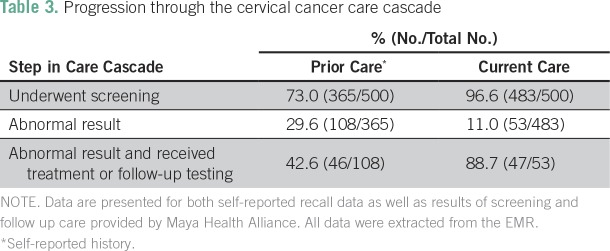
Progression through the cervical cancer care cascade

#### LTFU and the care cascade at MHA.

With MHA cytology results and documentation of follow-up care from the EMR (Table 3; Fig 1B), 96.6% (n = 483) of the 500 eligible individuals received screening, and 11.0% (53 of 483 women) had an abnormal result that required follow-up. Among these abnormal results, 7.7% were CIN lesions, whereas 92.3% were severe inflammation without precancerous lesions. A total of 88.7% (47 of 53 women) received follow-up testing or treatment (100% of those with precancerous lesions and 87.5% of those with severe inflammation), which yielded an effective LTFU of 11.3%.

### Structured Interviews

We interviewed 54 women who presented for screening in March 2013. We conducted 25 interviews in a Mayan language (n = 23, Kaqchikel; n = 2 K’iche’) and the rest in Spanish.

#### Knowledge about cervical cancer screening.

Forty-nine women (90.7%) reported prior knowledge of cervical cancer. However, few understood the etiology. For example, some (6.1%; three of 49 women) linked it to use of family planning methods or to multiparity (4.1%; two of 49 women). One woman explained:

“[T]he womb we have is for collecting illnesses. You’ve already gotten operated, you’ve already given birth to children…. That is where all of the illnesses come from.”

In addition, 16.3% (eight of 49 women) felt that cervical cancer was caused by a vaginal infection and described symptoms (discharge, bleeding, or pain), but none mentioned asymptomatic HPV infection. Finally, nearly all mentioned screening techniques, including cytology (98.1%; 53 of 54 women) and VIA (3.7%; two of 54 women).

#### Motivations for screening and social supports.

Most women (50 of 54 women, or 92.6%) presented for screening to promote wellness. For example, one remarked: “I want to know if I am well, to make sure I do not have sicknesses in my womb.” In addition, some (14.8%; eight of 54 women) reported concurrent gynecologic symptoms, such as vaginal discharge, and hoped screening could find the cause. For example, one stated that “I suffer frequently from many [vaginal] infections, I want to see if they could be from a cancer.”

When asked if anyone encouraged them to seek screening, 51.9% (28 of 54 women) reported that family members—often sisters or sisters-in-law (25%; seven of 28 women)—influenced their decision. Doctors, community, heath workers, and traditional midwives were other sources of influence. Eight (14.8%) of the 54 participants had family members or friends who had died as a result of cervical cancer. When asked if anyone had discouraged screening, eight (14.8%) of the 54 mentioned community-level gossip about ill effects of screening, but only one (1.9%) of the 54 interviewees had been specifically discouraged by a family member (her mother).

The majority of women (67%; 36 of 54 women) felt that they should independently make their own decisions for screening without permission from a spouse. They offered sentiments such as, “The woman herself [should decide], because it’s for her well-being,” or “The woman herself [should decide], because we are the ones who suffer.” At the same time, however, many reported restricted agency. Twenty-one (39%) of the 54 women reported asking permission from their spouses to be screened. However, some offered that they might get screened even without permission from their spouse, including one woman who noted that “It depends on the communication and the response. If he [my husband] is going to tell me yes, I ask him. If he is going to tell me no, I will do it without asking him.”

#### Other barriers and loss to follow-up.

Out-of-pocket costs were a common reason (20%; 11 of 54 women) for not seeking screening or for not repeating screening with the recommended frequency. One woman reported that “I have never done it. They asked me to do it before, but my husband did not have the money.” Others reported that travel to reach screening was prohibitive (9%; five of 54 women) or cited difficulties with missing work (9%; five of 54 women) or with arranging childcare (59%; 32 of 54 women). Regarding free-of-charge screening, 33.3% (18 of 54 women) reported that free public clinics only intermittently offered screening and that attending specific days was difficult.

When asked about factors that contribute to lack of engagement in care and LTFU, interviewees offered several reasons. Some (9%; five of 54 women) reported never receiving their screening results or not being offered follow-up for abnormal findings. Others expressed distrust in results: two (3.7%) of the 54 women cited stories in which acquaintances were diagnosed with cervical cancer after negative screens. One woman commented: “We don’t trust the health center, because the results never come. Or, what happened with one woman, she got a negative result but ended up with cancer.” Another reported: “If the test turns out positive, is anyone going to help you get treatment?”

## DISCUSSION

Knowledge about the cervical cancer screening and care continuum in Guatemala is still emerging, but what data exist suggest major shortcomings. For example, Guatemala’s Demographic Health Survey demonstrates suboptimal cervical cancer screening, especially for indigenous Maya women: only 43.5% report prior screening.^28^ In addition, in a recent study at the main cancer treatment facility in Guatemala, INCAN, only 35% of individuals with invasive cervical cancer completed recommended treatment and follow-up.^29^

Literature from other LMICs is similarly incomplete and demonstrates similar patterns. For example, there is widespread underutilization of screening services across many LMICs.^30,31^ In addition, the challenge of tracking patients after an abnormal first screening result has been well documented in multiple settings.^18,30,32^ Despite these reports, more systematic analysis of the continuum of cervical cancer care is infrequent in the literature from LMICs.^33,34^ This is in contrast to the infectious disease literature, which has used the concept of the care cascade to map flows and highlight critical steps at which care discontinuities or LTFU occur.^35-38^ Mapping the continuum of care has, for HIV and tuberculosis, led to successful systems-level interventions to retain individuals in care and improve outcomes.^39,40^

We mapped the cervical cancer screening care cascade, by using both self-reported (Table 3; Fig 1A) and programmatic screening data (Table 3; Fig 1B). Both mapping efforts demonstrated two obvious drop-off points along the continuum of care: individuals eligible for screening who were not screened and individuals who required retesting/treatment of abnormal results.

Quantitatively, the proportion of individuals who received screening in the MHA program was much higher than that of their prior reported screening rate. This likely represents, in part, selection bias, given that we included only individuals who had a screening intake form and, therefore, a presumed interest in screening. However, comparison of self-reported and observed LTFU rates for these individuals may provide insights into improvement care. For example, the proportion who received retesting/treatment was much higher in the MHA program than in those with prior reported screening (Table 3; Fig 1). This likely reflects the effectiveness of the nurse-led door-to-door case management strategy used to retain individuals in care. This is similar to a report from South Africa, where community health worker visits reduced LTFU after an abnormal screen.^18^

Another observation is that all patients at MHA with a CIN result successfully completed clinical treatment, and all cases of LTFU occurred in women with severe inflammation. There are two possible explanations. First, women with CIN may have been more motivated to follow up, given the immediate link with malignancy. Second, although treatment is offered directly after a CIN result, severe inflammation implies repetition of cervical cytology in 6 months, which adds more steps to the care cascade (Appendix Fig A1). Because the new HPV-based guidelines in Guatemala continue to recommend sequential repeat cytology,^15^ this vulnerable point for LTFU will not improve with modernization of screening and will require ongoing system-level interventions to improve retention in care.

Qualitative interviews highlighted several drivers of LTFU documented in the care cascade analysis. Regarding eligible women not screened, interviews identified misconceptions about risk factors. Some individuals believed that family planning increased risk, and others cited vaginal symptoms as their motivation for screening. Few were aware of the asymptomatic nature of most HPV infections. This argues for adaptation and implementation of evidence-based education strategies in Guatemala, such as the public marketing campaigns shown to improve screening in Honduras.^41^ Furthermore, cost of screening and transportation are two other barriers that were partly overcome by the MHA model, which brought services to rural communities for free. Another barrier identified was lack of support from male partners, and this should be a target for future interventions. Indeed, involvement of male partners in screening follow-up decreased LTFU in Uganda.^17^

Regarding the second major drop-off in care—LTFU at the retesting/treatment phase—interviewees described concerns about receiving screening results in a timely fashion, lack of resources for follow-up, and unreliable results (false negatives). These findings are obvious points for interventions to improve turnaround time, to better communicate follow-up plans, and to ensure formal linkages to definitive care when treatment is needed.^33,34^ The LTFU rate for the primary care MHA program was much lower than the self-reported historical rate (11.3 *v* 57.4%). The MHA program includes financial assistance for those who require follow-up care, care navigator assistance, and medical services provided by nurses fluent in Mayan languages.^38,39^ Future research should rigorously test whether these elements causally affect LTFU.

This study has important limitations. First, prior cervical cancer screening and care were self-reported. There may be underreporting of abnormal results or misclassification of treatments. Second, because the primary care program studied here offers comprehensive services—including family planning and prenatal care—alongside screening, findings may not be generalizable to larger-scale targeted screening programs. The EMR review identified patients on the basis of screening intake forms and thus represents a selected cohort; screening acceptance rates are likely higher, and LTFU likely lower, than in a sample that includes individuals not seeking screening.

In this article, we present an analysis of the care continuum of a cervical cancer screening and treatment program in rural Guatemala, in which screening was provided by a community-based organization and treatment, by a tertiary care cancer center. We identify two major transition points at which drop-off occurs: screening of eligible individuals and rescreening/treatment after first abnormal screen. Guatemala is in the process of transitioning toward HPV-based screening rather than the cytology-based programming studied here. However, our care cascade analysis shows that this new technology alone will not solve the systems-levels issues of poor screening uptake and LTFU. A research project underway at our site will develop a care cascade tool to measure LTFU prospectively. We encourage others to adopt the care cascade analysis paradigm to foster cross-site comparisons focused on improvement in the quality of cancer care in low-resource settings.
